# Vitamin D and Metabolic Syndrome in Working Age Subjects from an Obesity Clinic

**DOI:** 10.3390/nu15204354

**Published:** 2023-10-12

**Authors:** Luisella Vigna, Michela Carola Speciani, Amedea Silvia Tirelli, Francesca Bravi, Carlo La Vecchia, Caterina Conte, Francesca Gori

**Affiliations:** 1 Centro Obesità e Lavoro, Unità di Salute Occupazionale, Fondazione IRCCS Ca’ Granda, Ospedale Maggiore Policlinico, 20122 Milan, Italy; amtirel.st@gmail.com; 2 Laboratorio di Statistica Medica, Dipartimento di Scienze Cliniche e di Comunità, Biometria ed Epidemiologia “G.A. Maccacaro”, Università degli Studi di Milano, 20133 Milan, Italy; michela.speciani@unimi.it (M.C.S.); francesca.bravi@unimi.it (F.B.); carlo.lavecchia@unimi.it (C.L.V.); 3 Dipartimento di Promozione delle Scienze Umane e della Qualità della Vita, Università Telematica San Raffaele Roma, 00166 Rome, Italy; caterina.conte@uniroma5.it; 4 Dipartimento di Endocrinologia, Nutrizione e Malattie Metaboliche, IRCCS MultiMedica, Sesto S. Giovanni, 20900 Milan, Italy; 5 Dipartimento di Anestesia, Rianimazione ed Emergenza Urgenza, Fondazione IRCCS Ca’ Granda Ospedale Maggiore Policlinico, 20122 Milan, Italy; francesca.gori@policlinico.mi.it

**Keywords:** vitamin D, metabolic syndrome, overweight, obesity, workers

## Abstract

Serum vitamin D (VitD) levels have been inversely related with metabolic syndrome (MetS), although the direct impact of VitD is still debated. This study examined 879 subjects of working age from an obesity and occupational clinic in Milan, Italy. Among these participants, 316 had MetS, while 563 did not. A multiple logistic regression analysis was conducted to determine the odds ratios (ORs) and the corresponding 95% confidence intervals (CIs) for MetS in relation to serum VitD levels. After controlling for age, sex, leisure time physical activity, and body mass index (BMI), individuals with VitD levels between 20 and 29.9 ng/dL, or at least 30 ng/dL, had approximately half the risk of developing MetS (OR: 0.52, 95% CI: 0.32–0.86 and OR: 0.50, 95% CI: 0.25–0.99, respectively) compared to those with VitD levels below 10 ng/dL. This study presents further evidence of the beneficial effect of adequate VitD levels on the risk of MetS in a population of overweight/obese workers, even after adjusting for BMI. This study supports the importance of testing for and—if required—supplementing VitD in individuals with metabolic risk factors.

## 1. Introduction

Although several factors influence serum vitamin D (VitD) levels (geographical location [1], clothing habits [2], skin color [2], sunscreen use [2], and age [3,4]), sunlight exposure and dietary intake are the major sources of VitD in humans. VitD is synthesized in the skin, by the action of sunlight, starting from 7-dehydrocholesterol, of which cholesterol is a precursor [5]. It is also available in the diet from animal (cholecalciferol) and plant (ergocalciferol) foods. Regardless of the source, two hydroxylations are required, first in the liver and second in the kidney, for VitD to take up the biologically active form, known as 1,25(OH)_2_ VitD or calcitriol [6]. Hereafter, we will refer to 1,25(OH)_2_ VitD simply as VitD.

Endogenous production can account for up to 90% of total VitD in healthy individuals, and any activity that reduces exposure to sunlight adversely affects plasma VitD levels [7,8]. Consequently, factors and conditions that involve less exposure to sunlight, such as working indoors or overnight, can have a negative impact on VitD status, while the ones involving direct exposure to sunlight, such as daytime outdoor leisure time physical activity (LPA), can exert a positive impact. A systematic review of the literature, examining VitD levels in different occupations, identified shift workers, healthcare workers, and indoor workers to be at higher risk of developing VitD deficiency [9]. The workers less exposed to sunlight may have VitD deficiency, and this could negatively affect health. In fact, an inadequate VitD level has been linked to a number of diseases, such as metabolic disorders [7], including metabolic syndrome (MetS) [10,11].

MetS is characterized by hypertension, central obesity, and dyslipidemia. It is also known as insulin resistance syndrome because of the pivotal role that insulin resistance plays in its pathophysiology together with chronic inflammation [12,13]. Given its high prevalence and associated comorbidities and complications, MetS is considered a global epidemic [14]. Modifiable risk factors for Mets include unhealthy diet [15] and physical inactivity [16].

Studies conducted in Asia revealed associations between inadequate VitD levels and MetS, elevated waist circumference, hypertriglyceridemia, and low-density lipoprotein cholesterol [17,18]. Clinical investigations supported the link between VitD deficiency and MetS. Patients with VitD deficiency in a cardiology unit exhibited higher levels of various metabolic disease markers compared to those with adequate VitD levels [19]. Additionally, higher plasma concentrations of VitD have been associated with a decrease in systolic and diastolic blood pressure, potentially reducing the risk of hypertension [20]. Low levels of VitD may also contribute to obesity, through the effects of calcitriol on calcium absorption, lipolysis, and lipogenesis [21]. In previous studies, we investigated different aspects of the MetS and its associations with gender, psychosocial risk factors, and occupational determinants [22,23].

However, the relationship between VitD and MetS remains open to discussion [24]. The lack of long-term follow-up studies limits the ability to establish a causal relationship, so that the inverse relationship between body mass index (BMI) and VitD can be attributed to its fat solubility and consequent lower availability in individuals with higher adiposity [11,24]. Previous genetic studies have also challenged the direction of this association with unclear results [25,26].

The aim of this study is to further address the relationship between VitD and MetS, with particular reference to an employed and working age population from a referral clinic in Milan (Italy), taking into careful consideration the potential confounding role of body fat.

## 2. Materials and Methods

This cross-sectional study included 879 consecutive participants of a nutritional education program organized by the Obesity and Work Center, Occupational Medicine Department, Fondazione IRCCS Ca Granda, Ospedale Maggiore Policlinico, Italy, latitude 45.465454 N.

Participants were recruited during a periodic occupational examination program performed throughout the year (from May 2013 to April 2015) in consideration of seasonal variation in VitD status. Exclusion criteria were the presence of chronic diseases impairing the physiological process of production and metabolism of VitD, such as renal failure or hyperparathyroidism, and VitD or calcium supplementation. Upon entering the study, each participant underwent a clinical consultation during which all routine measurements, including anthropometrics, clinical anamnesis, and information on nutritional intakes and LPA, were collected.

We recorded LPA as never, sometimes (less than 2 h/week), and often (2 or more hours/week). BMI was obtained as the ratio between weight (kg) and squared height (m^2^) and categorized according to World Health Organization’s standard classification: participants were classified as affected by overweight when BMI was between 25.0 and 29.9 kg/m^2^, and by obesity when BMI was equal to 30 kg/m^2^ or greater. Obesity was further classified into 3 groups according to its severity (class I obesity: BMI between 30.0 and 34.9 kg/m^2^; class II obesity: BMI between 35.0 and 39.9 kg/m^2^; class III obesity: BMI greater or equal to 40.0 kg/m^2^). Blood pressure (both systolic and diastolic) and heart rate were also obtained. MetS was defined according to the 2005 U.S. National Cholesterol Education Program–Adult Treatment Panel III criteria [27] and considered prevalent at recruitment with the presence of at least 3 among: elevated waist circumference (greater than 102 cm in men, 88 cm in women); elevated triglycerides (greater than 150 mg/dL or therapy); reduced high-density lipoprotein (HDL) cholesterol (lower than 40 mg/dL in men, 50 mg/dL in women or therapy); elevated blood pressure (greater than 130 mmHg systolic blood pressure or 85 mmHg diastolic blood pressure or therapy); elevated fasting glucose (greater than 100 mg/dL or therapy).

According to the European Association for the Study of Obesity’s recommendations, an inclusive and respectful language was used, aiming to reduce any bias associated with the term “obesity” and stop the stigma that may come from labeling patients by their condition [28].

Routine biochemical parameters (e.g., glycemia, triglycerides, total and HDL cholesterol) were obtained via colorimetric and enzymatic assays run on automated platform Modular D (Roche, Basel, Switzerland) on the fasting serum sample. Plasma glycated hemoglobin was obtained using high-performance liquid chromatography. VitD status was evaluated as its circulating form 25(OH)D, using DiaSorin’s 25-OH VitD TOTAL competitive chemiluminescence immunoassay on an automated LIASON instrument (Saluggia, Italy).

Subjects were classified as VitD severely deficient (<10 ng/mL), moderately deficient (10–19 ng/mL), or insufficient (20–29 ng/mL), following the 2011 clinical practice guidelines by the Endocrine Society [8], with 30 ng/mL being considered as the minimum sufficient level.

Unconditional multiple logistic regression models were fitted to compute the odds ratios (OR) of MetS and the corresponding 95% confidence intervals (CIs), according to serum VitD levels, with severe deficiency as the reference category. The model included terms for sex (males, females), age (≤35, >36 and ≤45, >45 and ≤50, >50 and ≤55, >55 years), BMI (<30, ≥30 and <35, ≥35 and <40, ≥40 kg/m^2^), and LPA (never, sometimes, often) as adjustment variables. Because of the presence of only 15 normal weight (over 18.5 and below 25 kg/m^2^ of BMI) subjects, the normal weight and the overweight categories were combined.

The complete model was also fitted across strata of sex (males or female), age (<50 or ≥50 years old), and BMI (<35 or ≥35 kg/m^2^) after testing for heterogeneity through a likelihood ratio test between models with and without the interaction term, together with a crude model and a model including all terms of the complete one but BMI, when appropriate.

Tests were considered statistically significant when the *p*-value was <0.05. Statistical analyses were carried out using R [29], version 4.2.0.

Ethical approval for the project was granted by the Local Ethics Committee (study registration number: 1370). All subjects signed an informed consent form to participate in the study.

## 3. Results

There were 316 subjects with and 563 without MetS. Participants had a mean age of 45 years (range: 18–60; standard deviation: 10.5), a mean BMI of 32.5 kg/m^2^ (range: 22.5–59.1; standard deviation: 5.4), a mean glycated hemoglobin of 37.7 mmol/mol (range: 23–57; standard deviation: 5.1), and a mean fasting glucose of 93 mg/dL (range: 69–165; standard deviation: 12). Mean serum VitD was 16.6 ng/mL (range: 3.0–69.5; standard deviation: 8.7). The majority (76%) of the subjects were women.

Table 1 gives the distribution of the study population according to selected variables, overall and in subjects with or without MetS. Subjects with MetS were older, had higher BMI, higher glycated hemoglobin, and lower VitD levels.

Table 2 shows the multivariable ORs and the corresponding 95% CIs of MetS for each level of serum VitD compared to the lowest one (<10 ng/dL). Compared to the severe deficiency category, ORs of MetS were 0.52 (95% CI: 0.32–0.86) for the VitD insufficiency category and 0.50 (95% CI: 0.25–0.99) for the sufficiency category. The inverse trend in risk between serum VitD levels and MetS was significant (p-trend: 0.007).

Table 3 shows stratified analyses according to three different models. Tests for heterogeneity among strata of sex, age, and BMI were non-significant in the complete models. Still, the inverse relation with serum VitD levels was apparently stronger in males, in subjects under 50, and in subjects with a BMI below 35 kg/m^2^ when the complete models were fitted. This is especially true across strata of age, where a favorable effect of higher levels of serum VitD was present in subjects below 50 years of age but not evident in subjects aged 50 or more. The confounding role of BMI on the relation between VitD and MetS was particularly noticeable among females: OR of MetS was 0.40 (95% CI: 0.19–0.85) for VitD ≥ 30 ng/mL compared to VitD below 10 ng/mL when BMI was not included in the model, and 0.69 (95% CI: 0.32–1.52) when the complete model (including BMI as an adjustment variable) was fitted.

## 4. Discussion

Given the involvement of VitD in several aspects of human health [30], numerous authors addressed the possible relationship between VitD and MetS [10]. We considered a worker population affected by overweight or obesity as some specific occupations were reported to have a higher risk of developing MetS [31]. Our results indicate that subjects with VitD levels above 20 ng/dL had about half the risk of MetS compared to those with a severe deficiency (<10 ng/dL).

Several studies have shown that populations worldwide, including those located in sunny areas, are at risk of developing VitD deficiency [32,33]. In fact, a high prevalence of VitD deficiency among a Southern Italian population was also found despite the low latitude [34]. Moreover, some vulnerable demographic groups of the population, including workers employed in specific occupations, often receive only minimal exposure to sunlight, and, therefore, their risk of developing VitD deficiency increases [35,36,37,38].

A study similar to ours was conducted with the aim of quantifying the association between serum VitD levels, the number of MetS components, and insulin resistance in the Canadian population as they are very common conditions in North America [39].

Several studies have reported similar findings, showing an inverse relationship between serum VitD and different human diseases, including those related to insulin resistance, such as diabetes, obesity, and cardiovascular disease [35,40,41]. Although this could be due to the presence of its receptors in many cells, including immune and parathyroid cells, pancreatic β cells, and endothelium, the specific mechanisms underlying this inverse relationship remain to be understood. The negative relation between BMI and blood concentration of VitD [42] could be explained by the reduced bioavailability of VitD itself, which accumulates in adipose tissue, and by the lower exposure to sunlight, due to the sedentary lifestyle of subjects suffering from obesity [43]. Furthermore, a central role of VitD in the regulation of parathyroid hormone (PTH) levels has been proposed, and a state of hypovitaminosis D could cause an increase in its levels which, in turn, would lead to an accumulation of calcium ions in adipocytes, stimulating lipogenesis with an avoidable growth of body mass [44]. Recently, in a study of over 42,000 subjects, obesity was identified as a causal risk factor influencing approximately one third of recorded cases of VitD deficiency. Therefore, obesity itself could be the cause of the increasingly widespread hypovitamonosis D in the population [8].

There are extensive studies indicating hypovitaminosis D as a risk factor for type 2 diabetes [45,46,47,48,49,50,51] and that supplementation can be a useful intervention [52,53]. Results from short-term studies suggest that the presence of VitD receptors on pancreatic β-cells, adipocite, and musculoskeletal cells could lead to low VitD levels to impair the conversion of pro-insulin to insulin by the β-cells [4,54,55]. This could be an explanation for the role that VitD plays in insulin secretion and sensitivity, which are critical in the development of MetS [11]. VitD regulates blood pressure by acting both on smooth muscle and endothelial cells. Several cardiovascular risk factors and an increased risk of incidence and mortality from cardiovascular diseases have been associated with hypovitaminosis D due to the abnormal production of oxide nitric, activation of the renin–angiotensin–aldosterone pathways, or through alteration of oxidative balance or anti-inflammatory systems [56]. A systematic review and meta-analysis demonstrated that VitD can inhibit the expression of serum C-reactive protein, tumor necrosis factor-α, and the production of oxidative stress markers, such as malondialdehyde [57]. Although it was concluded that VitD can be considered a valid instrument for alleviating inflammation and oxidative stress, the meta-analysis also highlighted that it has no effect on specific markers, such as interleukin-6, glutathione, or molecules that constitute the total antioxidant capacity [57]. All these mentioned functions of VitD have an impact on human metabolic health and are part of the pathophysiology of MetS [13,58].

Only 9% of our sample reached adequate levels of serum VitD. Given its exposure to the Mediterranean diet, the Italian population is commonly considered to be able to meet a sufficient daily intake of VitD. In our population of indoor workers exhibited widespread suboptimal and deficient VitD levels instead. In these subjects, average daily intake of VitD is inadequate for maintaining sufficient serum levels, and probably not meeting the optimal 5–15 mcg suggested as dietary reference intakes [59]. Indeed, as previously shown [40], even during the period with the highest VitD levels (autumn), values still remained below the optimal range compared to the reference guidelines [60,61], in agreement with previous data on Northern populations [62] and the ranges for the appropriate cut-off for VitD optimal concentrations [63,64,65]. Seventy percent of our population was also affected by obesity of varying degrees. With regard to obesity, as mentioned above, hypovitaminosis D in affected subjects could at least be partially explained by VitD storage in adipose tissue [43], and low VitD leads to both elevated PTH and increased calcium in adipocytes with influence on lipogenesis and adiposity [66].

We could not exclude reverse causation, since overweight and obesity may have an impact on both VitD levels and MetS risk. However, we carefully adjusted for BMI, hence our findings could not be attributed to reverse causation only. Although a relationship between VitD and components of MetS has been widely discussed VitD deficiency may be secondary to the metabolic changes in MetS subjects [67].

In addition, we could not consider VitD supplementation. However, given the attention that VitD is receiving in metabolic health, we have no reason to suppose that patients with MetS should receive less VitD supplementation compared to the ones without MetS diagnosis. We also were unable to consider serum VitD seasonality, with patients tested in summer to early autumn possibly having higher levels of VitD due to sun exposure. However, the enrolling and testing of our study population were spread throughout the whole year, thus reducing this bias.

The strengths of our research are the large sample size, together with the characteristics of our study population. Particularly, we were able to assess the potential role of VitD in a population with high BMI and different levels of obesity. Moreover, our data derive from a standardized and specialized clinical practice. Another strength is that we were able to consider LPA, a potential confounder for its effect on both metabolic health and VitD levels, when outdoor LPA is practiced.

## 5. Conclusions

The present study demonstrates an association between serum VitD level and MetS in an Italian working population affected by overweight or obesity, and that the risk of MetS increases with decreasing serum VitD concentration, even when LPA and BMI were taken into account. Although prospective studies are still useful to assess and quantify the direct impact of VitD on MetS over time, our results advise towards serum VitD testing and consequent supplementation when needed in working age overweight populations.

## Figures and Tables

**Table 1:** Distribution of the 879 subjects included in our study, overall and with/without metabolic syndrome (MetS), according to selected variables including anthropometric/physical/biochemical NCEP/ATP III metabolic syndrome diagnosis criteria.

Population Characteristics	Overall Population N (%)	Subjects without MetS N (%)	Subjects with MetS N (%)
Age *			
<35	157 (17.9)	133 (23.6)	24 (7.6)
35–39	85 (9.7)	61 (10.8)	24 (7.6)
40–44	128 (14.6)	83 (14.7)	45 (14.2)
45–49	166 (18.9)	102 (18.1)	64 (20.3)
50–54	162 (18.4)	87 (15.5)	75 (23.7)
≥55	181 (20.6)	97 (17.2)	84 (26.6)
Sex *			
males	212 (24.1)	96 (17.1)	116 (36.7)
females	667 (75.9)	467 (82.9)	200 (63.3)
Body mass index *			
<30 kg/m^2^	256 (29.1)	219 (38.9)	37 (11.7)
≥30 and <35 kg/m^2^	333 (37.9)	212 (37.7)	121 (38.3)
≥35 and <40 kg/m^2^	180 (20.5)	81 (14.4)	99 (31.3)
≥40 kg/m^2^	110 (12.5)	51 (9.1)	59 (18.7)
Glycated hemoglobin *			
≤38 mmol/mol	511 (58.1)	386 (68.6)	125 (39.6)
>38 and <48 mmol/mol	336 (38.2)	170 (30.2)	166 (52.5)
≥48 mmol/mol	32 (3.6)	7 (1.2)	25 (7.9)
Antidiabetic drugs *			
no	795 (90.4)	537 (95.4)	258 (81.6)
yes	84 (9.6)	26 (4.6)	58 (18.4)
**Leisure time physical activity**			
never	592 (67.3)	373 (66.3)	219 (69.3)
sometimes	235 (26.7)	155 (27.5)	80 (25.3)
often	52 (5.9)	35 (6.2)	17 (5.4)
Serum 25-OH vitamin D levels *			
<10 ng/mL	157 (17.9)	79 (14.0)	78 (24.7)
≥10 and <20 ng/mL	418 (47.6)	258 (45.8)	160 (50.6)
≥20 and< 30 ng/mL	225 (25.6)	166 (29.5)	59 (18.7)
≥30 ng/mL	79 (9.0)	60 (10.7)	19 (6.0)
**Metabolic Syndrome Biochemical/Physical/Anthropometric Diagnostic Criteria**			
Waist circumference *			
normal	207 (23.5)	190 (33.7)	17 (5.4)
high ^1^	672 (76.5)	373 (66.3)	299 (94.6)
Triglycerides *			
<150 mg/dL	688 (78.3)	508 (90.2)	180 (57.0)
≥150 mg/dL	191 (21.7)	55 (9.8)	136 (43.0)
HDL cholesterol *			
low ^2^	184 (20.9)	55 (9.8)	129 (40.8)
normal	695 (79.1)	508 (90.2)	187 (59.2)
Systolic blood pressure *			
<130 mmHg	567 (64.5)	421 (74.8)	146 (46.2)
≥130 mmHg	312 (35.5)	142 (25.2)	170 (53.8)
Diastolic blood pressure *			
<85 mmHg	669 (76.1)	459 (81.5)	210 (66.5)
≥85 mmHg	210 (23.9)	104 (18.5)	106 (33.5)
High blood pressure ^3,^*			
no	515 (58.6)	388 (68.9)	127 (40.2)
yes	364 (41.4)	175 (31.1)	189 (59.8)
Fasting glucose *			
<100 mg/dL	682 (77.6)	508 (90.2)	174 (55.1)
≥100 mg/dL	197 (22.4)	55 (9.8)	142 (44.9)

^1^ Defined as ≥102 cm in men or ≥88 cm in women. ^2^ Defined as <40 mg/dL in men or <50 mg/dL in women. ^3^ Defined as systolic blood pressure ≥ 130 mmHg or diastolic blood pressure ≥ 85 mmHg. * Chi-square for the distribution of subjects with/without metabolic syndrome according to the population characteristic, *p*-value < 0.0001. Abbreviations: HDL, high-density lipoprotein; NCEP-ATP III, National Cholesterol Education Program Adult Treatment Panel III.

**Table 2:** Odds ratios (ORs) and the corresponding 95% confidence intervals (CIs) of metabolic syndrome (MetS) according to serum vitamin D levels.

	Overall Population (N: 879)			
Serum Vitamin D levels	Subjects without MetS N (%)	Subjects with MetS N (%)	OR (95% CI) ^a^	*p*-Trend
<10 ng/mL	79 (14.0)	78 (24.7)	1 (reference)	
≥10 and <20 ng/mL	258 (45.8)	160 (50.6)	0.72 (0.48–1.09)	
≥20 and <30 ng/mL	166 (29.5)	59 (18.7)	**0.52 (0.32–0.86)**	
≥30 ng/mL	60 (10.7)	19 (6.8)	**0.50 (0.25–0.99)**	**0.007**

^a^ Estimated from multiple unconditional logistic regression models including terms for sex (males, females), categories of age (≤35, >36 and ≤45, >45 and ≤50, >50 and ≤55, >55), categories of body mass index (<30, ≥30 and <35, ≥35 and <40, ≥40 kg/m^2^), and leisure time physical activity levels (never, sometimes, often). Results reported in bold when *p*-value < 0.05.

**Table 3:** Odds ratios (ORs) and the corresponding 95% confidence intervals (CIs) of metabolic syndrome (MetS) according to levels of serum vitamin D (VitD) across selected strata.

	Subjects without/with MetS N/N	OR (95% CI) ^a^	OR (95% CI) ^b^	OR (95% CI) ^c^	Subjects without/with MetS N/N	OR (95% CI) ^a^	OR (95% CI) ^b^	OR (95% CI) ^c^
STRATA	SEX							
VitD (ng/mL)	Males				Females			
<10	13/31	1 (reference)	1 (reference)	1 (reference)	66/47	1 (reference)	1 (reference)	1 (reference)
≥10 and <20	45/51	0.48 (0.22–1.02)	**0.37 (0.16–0.87)**	**0.38 (0.15–0.93)**	213/109	0.71 (0.49–1.03)	0.72 (0.46–1.14)	0.88 (0.54–1.42)
≥20 and <30	26/28	0.45 (0.20–1.05)	**0.33 (0.13–0.83)**	0.43 (0.16–1.17)	140/31	**0.31 (0.18–0.53)**	**0.33 (0.19–0.58)**	**0.53 (0.29–0.95)**
≥30	12/6	**0.21 (0.06–0.68)**	**0.15 (0.04–0.55)**	**0.18 (0.04–0.72**)	48/13	**0.38 (0.19–0.78)**	**0.40 (0.19–0.85)**	0.69 (0.32–1.52)
**p-trend**		**0.011**	**0.004**	**0.031**		**<0.001**	**<0.001**	0.060
**p-hetero**						0.220	**<0.001**	0.180
**STRATA**	**AGE**							
**VitD (** **ng/mL)**	**<50**				**≥50**			
<10	49/43	1 (reference)	1 (reference)	1 (reference)	30/35	1 (reference)	1 (reference)	1 (reference)
≥10 and <20	171/80	**0.53 (0.33–0.87)**	**0.55 (0.33–0.92)**	0.61 (0.36–1.03)	87/80	0.79 (0.44–1.40)	0.81 (0.45–1.46)	1.02 (0.54–1.92)
≥20 and <30	116/27	**0.27 (0.15–0.48)**	**0.27 (0.15–0.50)**	**0.35 (0.18–0.65)**	50/32	0.55 (0.28–1.06)	0.53 (0.27–1.04)	1.00 (0.46–2.16)
≥30	43/7	**0.19 (0.08–0.46)**	**0.21 (0.08–0.51)**	**0.26 (0.10–0.68)**	17/12	0.61 (0.25–1.47)	0.56 (0.23–1.40)	1.26 (0.45–3.54)
**p-trend**		**<0.001**	**<0.001**	**<0.001**		0.080	0.055	0.761
**p-hetero**						0.206	0.324	0.165
**STRATA**	**BMI**							
**VitD (** **ng/mL)**	**<35 kg/m^2^**				**≥35 kg/m^2^**			
<10	48/34	1 (reference)	-	1 (reference)	31/44	1 (reference)	-	1 (reference)
≥10 and <20	189/73	**0.55 (0.33–0.91)**	-	**0.51 (0.29–0.88)**	69/87	0.89 (0.51–1.55)	-	0.90 (0.49–1.65)
≥20 and <30	144/36	**0.35 (0.20–0.62)**	-	**0.34 (0.18–0.62)**	22/23	0.74 (0.35–1.55)	-	0.85 (0.37–1.94)
≥30	50/15	**0.42 (0.21–0.87)**	-	**0.40 (0.18–0.86)**	10/4	**0.28 (0.08–0.98)**	-	0.29 (0.07–1.19)
**p-trend**		**0.002**	-	**0.002**		0.073	-	0.186
**p-hetero**						0.247	-	0.230

^a^ Estimated from simple unconditional logistic regression models. ^b^ Estimated from multiple unconditional logistic regression models including terms for sex (males, females), categories of age (≤35, >36 and ≤45, >45 and ≤50, >50 and ≤55, >55), and leisure time physical activity levels (never, sometimes, often) when appropriate. ^c^ Estimated from the previous model with the further addition of categories of body mass index (<30, ≥30 and <35, ≥35 and <40, ≥40 kg/m^2^) when appropriate. Results reported in bold when *p*-value < 0.05. Abbreviations: BMI, body mass index.

## Data Availability

The data presented in this paper will be available from the corresponding author upon reasonable request.

## References

[1] Leal A.C.G.B., Corrêa M.P., Holick M.F., Melo E.V., Lazaretti-Castro M. (2021). Sun-induced production of vitamin D throughout 1 year in tropical and subtropical regions: Relationship with latitude, cloudiness, UV-B exposure and solar zenith angle. Photochem. Photobiol. Sci..

[2] Holick M.F. (2023). The One-Hundred-Year Anniversary of the Discovery of the Sunshine Vitamin D3: Historical, Personal Experience and Evidence-Based Perspectives. Nutrients.

[3] Holick M.F. (2020). A call for action: Standard of care guidelines to assess vitamin D status are needed for patients with hip fracture. Am. J. Clin. Nutr..

[4] Schmitt E.B., Nahas-Neto J., Bueloni-Dias F., Poloni P.F., Orsatti C.L., Petri Nahas E.A. (2018). Vitamin D deficiency is associated with metabolic syndrome in postmenopausal women. Maturitas.

[5] Knuschke P. (2021). Sun Exposure and Vitamin D. Curr. Probl. Dermatol..

[6] Borel P., Caillaud D., Cano N.J. (2015). Vitamin D bioavailability: State of the art. Crit. Rev. Food Sci. Nutr..

[7] Holick M.F. (2007). Vitamin D deficiency. N. Engl. J. Med..

[8] Holick M.F., Binkley N.C., Bischoff-Ferrari H.A., Gordon C.M., Hanley D.A., Heaney R.P., Murad M.H., Weaver C.M., Endocrine Society (2011). Evaluation, treatment, and prevention of vitamin D deficiency: An Endocrine Society clinical practice guideline. J. Clin. Endocrinol. Metab..

[9] Sowah D., Fan X., Dennett L., Hagtvedt R., Straube S. (2017). Vitamin D levels and deficiency with different occupations: A systematic review. BMC Public. Health.

[10] Mansouri M., Abasi R., Nasiri M., Sharifi F., Vesaly S., Sadeghi O., Rahimi N., Sharif N.A. (2018). Association of vitamin D status with metabolic syndrome and its components: A cross-sectional study in a population of high educated Iranian adults. Diabetes Metab. Syndr..

[11] Melguizo-Rodríguez L., Costela-Ruiz V.J., García-Recio E., De Luna-Bertos E., Ruiz C., Illescas-Montes R. (2021). Role of Vitamin D in the Metabolic Syndrome. Nutrients.

[12] Meshkani R., Adeli K. (2009). Hepatic insulin resistance, metabolic syndrome and cardiovascular disease. Clin. Biochem..

[13] Alberti K.G., Eckel R.H., Grundy S.M., Zimmet P.Z., Cleeman J.I., Donato K.A., Fruchart J.-C., James W.P.T., Loria C.M., Smith S.C. (2009). Harmonizing the metabolic syndrome: A joint interim statement of the international diabetes federation task force on epidemiology and prevention; national heart, lung, and blood institute; American heart association; world heart federation; international atherosclerosis society; and international association for the study of obesity. Circulation.

[14] Saklayen M.G. (2018). The Global Epidemic of the Metabolic Syndrome. Curr. Hypertens. Rep..

[15] García S., Pastor R., Monserrat-Mesquida M., Álvarez-Álvarez L., Rubín-García M., Martínez-González M.Á., Salas-Salvadó J., Corella D., Goday A., Martínez J.A. (2023). Metabolic syndrome criteria and severity and carbon dioxide (CO_2_) emissions in an adult population. Glob. Health.

[16] Bovolini A., Garcia J., Andrade M.A., Duarte J.A. (2021). Metabolic Syndrome Pathophysiology and Predisposing Factors. Int. J. Sports Med..

[17] Liu L., Cao Z., Lu F., Liu Y., Lv Y., Qu Y., Gu H., Li C., Cai J., Ji S. (2020). Vitamin D Deficiency and Metabolic Syndrome inElderly Chinese Individuals: Evidence from CLHLS. Nutr. Metab..

[18] Lee S.J., Lee E.Y., Lee J.H., Kim J.E., Kim K.J., Rhee Y., Kim H.C., Youm Y., Kim C.O. (2019). Associations of Serum 25-Hydroxyvitamin D with Metabolic Syndrome and Its Components in Elderly Men and Women: The Korean Urban RuralElderly Cohort Study. BMC Geriatr..

[19] Pott-Junior H., Nascimento C.M.C., Costa-Guarisco L.P., Gomes G.A.O., Gramani-Say K., Orlandi F.S., Gratão A.C.M., Orlandi A.A.D.S., Pavarini S.C.I., Vasilceac F.A. (2020). Vitamin D Deficient Older Adults Are More Prone to Have Metabolic Syndrome, but Not to a Greater Number of Metabolic Syndrome Parameters. Nutrients.

[20] Vimaleswaran K.S., Cavadino A., Berry D.J., Jorde R., Dieffenbach A.K., Lu C., Alves A.C., Heerspink H.J.L., Tikkanen E. (2014). Association of Vitamin D Status with Arterial Blood Pressure and Hypertension Risk: A Mendelian Randomisation Study. Lancet Diabetes Endocrinol..

[21] Ghaderi A., Banafshe H.R., Motmaen M., Rasouli-Azad M., Bahmani F., Asemi Z. (2017). Clinical Trial of the Effects of Vitamin D Supplementation on Psychological Symptoms and Metabolic Profiles in Maintenance Methadone Treatment Patients. Prog. Neuropsychopharmacol. Biol. Psychiatry.

[22] Vigna L., Vassalle C., Tirelli A.S., Gori F., Tomaino L., Sabatino L., Bamonti F. (2017). Gender-related association between uric acid, homocysteine, γ-glutamyltransferase, inflammatory biomarkers and metabolic syndrome in subjects affected by obesity. Biomark. Med..

[23] Vigna L., Brunani A., Brugnera A., Grossi E., Compare A., Tirelli A.S., Conti D.M., Agnelli G.M., Andersen L.L., Buscema M. (2019). Determinants of metabolic syndrome in obese workers: Gender differences in perceived job-related stress and in psychological characteristics identified using artificial neural networks. Eat. Weight. Disord..

[24] Vimaleswaran K.S., Berry D.J., Lu C., Tikkanen E., Pilz S., Hiraki L.T., Cooper J.D., Dastani Z., Li R., Houston D.K. (2013). Causal relationship between obesity and vitamin D status: Bi-directional Mendelian randomization analysis of multiple cohorts. PLoS Med..

[25] Xu Y., Zhou Y., Liu J., Wang C., Qu Z., Wei Z., Zhou D. (2020). Genetically increased circulating 25(OH)D level reduces the risk of type 2 diabetes in subjects with deficiency of vitamin D: A large-scale Mendelian randomization study. Medicine.

[26] Chen C., Chen Y., Weng P., Xia F., Li Q., Zhai H., Wang N., Lu Y. (2019). Association of 25-hydroxyvitamin D with cardiometabolic risk factors and metabolic syndrome: A mendelian randomization study. Nutr. J..

[27] Grundy S.M., Cleeman J.I., Daniels S.R., Donato K.A., Eckel R.H., Franklin B.A., Gordon D.J., Krauss R.M., Savage P.J., Smith S.C. (2005). American Heart Association. National Heart, Lung, and Blood Institute: Diagnosis and management of the metabolic syndrome: An American Heart Association/National Heart, Lung, and Blood Institute scientific statement. Circulation.

[28] Rubino F., Puhl R.M., Cummings D.E., Eckel R.H., Ryan D.H., Mechanick J.I., Nadglowski J., Ramos Salas X., Schauer P.R., Twenefour D. (2020). Joint international consensus statement for ending stigma of obesity. Nat. Med..

[29] R Development Core Team (2022). R: A Language and Environment for Statistical Computing.

[30] Heaney R.P. (2008). Vitamin D in health and disease. Clin. J. Am. Soc. Nephrol..

[31] Sooriyaarachchi P., Jayawardena R., Pavey T., King N.A. (2022). Shift work and the risk for metabolic syndrome among healthcare workers: A systematic review and meta-analysis. Obes. Rev..

[32] Gonzalez G., Alvarado J.N., Rojas A., Navarrete C., Velasquez C.G., Arteaga E. (2007). High prevalence of vitamin D deficiency in Chilean healthy postmenopausal women with normal sun exposure: Additional evidence for a worldwide concern. Menopause.

[33] van der Meer I.M., Middelkoop B.J., Boeke A.J., Lips P. (2011). Prevalence of vitamin D deficiency among Turkish, Moroccan, Indian and sub-Sahara African populations in Europe and their countries of origin: An overview. Osteoporos. Int..

[34] Capuano R., Marchese F., Sica R., Capuano E., Manilia M., Iannone A.G., D’Ambrosio A., Bisecco A., Tedeschi G., Gallo A. (2021). Epidemiologic Data of Vitamin D Deficiency and Its Implication in Cardio-Cerebrovascular Risk in a Southern Italian Population. J. Nutr. Metab..

[35] Romano A., Vigna L., Belluigi V., Conti D.M., Barberi C.E., Tomaino L., Consonni D., Riboldi L., Tirelli A.S., Andersen L.L. (2015). Shift work and serum 25-OH vitamin D status among factory workers in Northern Italy: Cross-sectional study. Chronobiol. Int..

[36] Kwon S.I., Son J.S., Kim Y.O., Chae C.H., Kim J.H., Kim C.W., Park H.O., Lee J.H., Jung J.I. (2015). Association between serum vitamin D and depressive symptoms among female workers in the manufacturing industry. Ann. Occup. Environ. Med..

[37] Roomi M.A., Farooq A., Ullah E., Lone K.P. (2015). Hypovitaminosis D and its association with lifestyle factors. Pak. J. Med. Sci..

[38] Xiang F., Jiang J., Li H., Yuan J., Yang R., Wang Q., Zhang Y. (2013). High prevalence of vitamin D insufficiency in pregnant women working indoors and residing in Guiyang, China. J. Endocrinol. Investig..

[39] Brenner D.R., Arora P., Garcia-Bailo B., Wolever T.M., Morrison H., El-Sohemy A., Karmali M., Badawi A. (2011). Plasma vitamin D levels and risk of metabolic syndrome in Canadians. Clin. Investig. Med..

[40] Vigna L., Cassinelli L., Tirelli A.S., Felicetta I., Napolitano F., Tomaino L., Mutti M., Barberi C.E., Riboldi L. (2017). 25(OH)D Levels in Relation to Gender, Overweight, Insulin Resistance, and Inflammation in a Cross-Sectional Cohort of Northern Italian Workers: Evidence in Support of Preventive Health Care Programs. J. Am. Coll. Nutr..

[41] Szymczak-Pajor I., Śliwińska A. (2019). Analysis of Association between Vitamin D Deficiency and Insulin Resistance. Nutrients.

[42] Earthman C.P., Beckman L.M., Masodkar K., Sibley S.D. (2012). The link between obesity and 25-hydroxyvitamin D concentrations: Considerations and implications. Int. J. Obes..

[43] Wortsman J., Matsuoka L.Y., Chen T.C., Lu Z., Holick M.F. (2000). Decreased bioavailability of vitamin D in Obesity. Am. J. Clin. Nutr..

[44] Snijder M.B., van Dam R.M., Visser M., Deeg D.J.H., Dekker J.M., Bouter L.M., Seidell J.C., Lips P. (2005). Adiposity in relation to vitamin D status and parathyroid hormone levels: A population-based study in older men and women. J. Clin. Endocrinol. Metab..

[45] Song Y., Wang L., Pittas A.G., Del Gobbo L.C., Zhang C., Manson J.E., Hu F.B. (2013). Blood 25-hydroxy vitamin D levels and incident type 2 diabetes: A meta-analysis of prospective studies. Diabetes Care.

[46] Pittas A.G., Nelson J., Mitri J., Hillmann W., Garganta C., Nathan D.M., Hu F.B., Dawson-Hughes B. (2012). Diabetes Prevention Program Research Group. Plasma 25-hydroxyvitamin D and progression to diabetes in patients at risk for diabetes: An ancillary analysis in the Diabetes Prevention Program. Diabetes Care.

[47] Barengolts E., Manickam B., Eisenberg Y., Akbar A., Kukreja S., Ciubotaru I. (2015). Effect of high-dose vitamin D repletion on glycemic control in African American males with prediabetes and hypovitaminosis D. Endocr. Pract..

[48] Kuchay M.S., Laway B.A., Bashir M.I., Wani A.I., Misgar R.A., Shah Z.A. (2015). Effect of vitamin D supplementation on glycemic parameters and progression of prediabetes to diabetes: A 1-year, open-label randomized study. Indian J. Endocrinol. Metab..

[49] Jorde R., Sollid S.T., Svartberg J., Schirmer H., Joakimsen R.M., Njølstad I., Fuskevåg O.M., Figenschau Y., Hutchinson M.Y. (2016). Vitamin D 20,000 IU per Week for Five Years Does Not Prevent Progression From Prediabetes to Diabetes. J. Clin. Endocrinol. Metab..

[50] Niroomand M., Fotouhi A., Irannejad N., Hosseinpanah F. (2019). Does high-dose vitamin D supplementation impact insulin resistance and risk of development of diabetes in patients with pre-diabetes? A double-blind randomized clinical trial. Diabetes Res. Clin. Pract..

[51] Pittas A.G., Dawson-Hughes B., Sheehan P., Ware J.H., Knowler W.C., Aroda V.R., Brodsky I., Ceglia L., Chadha C., Chatterjee R. (2019). D2d Research Group. Vitamin D Supplementation and Prevention of Type 2 Diabetes. N. Engl. J. Med..

[52] Lu L., Bennett D.A., Millwood I.Y., Parish S., McCarthy M.I., Mahajan A., Lin X., Bragg F., Guo Y., Holmes M.V. (2018). Association of vitamin D with risk of type 2 diabetes: A Mendelian randomisation study in European and Chinese adults. PLoS Med..

[53] Pittas A.G., Lau J., Hu F.B., Dawson-Hughes B. (2007). The role of vitamin D and calcium in type 2 diabetes. A systematic review and meta-analysis. J. Clin. Endocrinol. Metab..

[54] Chiu K.C., Chu A., Go V.L., Saad M.F. (2004). Hypovitaminosis D is associated with insulin resistance and beta cell dysfunction. Am. J. Clin. Nutr..

[55] Mitri J., Dawson-Hughes B., Hu F.B., Pittas A.G. (2011). Effects of vitamin D and calcium supplementation on pancreatic β cell function, insulin sensitivity, and glycemia in adults at high risk of diabetes: The Calcium and Vitamin D for Diabetes Mellitus (CaDDM) randomized controlled trial. Am. J. Clin. Nutr..

[56] de la Guía-Galipienso F., Martínez-Ferran M., Vallecillo N., Lavie C.J., Sanchis-Gomar F., Pareja-Galeano H. (2021). Vitamin D and cardiovascular health. Clin. Nutr..

[57] Moslemi E., Musazadeh V., Kavyani Z., Naghsh N., Shoura S.M.S., Dehghan P. (2022). Efficacy of vitamin D supplementation as an adjunct therapy for improving inflammatory and oxidative stress biomarkers: An umbrella meta-analysis. Pharmacol. Res..

[58] Illescas-Montes R., Melguizo-Rodríguez L., Ruiz C., Costela-Ruiz V.J. (2019). Vitamin D and autoimmune diseases. Life Sci..

[59] Institute of Medicine (US) Committee on Use of Dietary Reference Intakes in Nutrition Labeling (2003). Dietary Reference Intakes: Guiding Principles for Nutrition Labeling and Fortification.

[60] Grober U., Reichrath J., Holick M.F. (2015). Live longer with vitamin D?. Nutrients.

[61] Bouillon R., Van Schoor N.M., Gielen E., Boonen S., Mathieu C., Vander Schueren D., Lips P. (2013). Optimal vitamin D status: A critical analysis on the basis of evidence-based medicine. J. Clin. Endocrinol. Metab..

[62] Chao Y.S., Brunel L., Faris P., Veugelers P.J. (2013). Vitamin D status of Canadians employed in northern latitudes. Occup. Med..

[63] Heaney R.P. (2015). Screening for vitamin D deficiency: Is the goal disease prevention or full nutrient repletion?. Ann. Intern. Med..

[64] Perna L., Haug U., Schöttker B., Müller H., Raum E., Jansen E.H., Brenner H. (2012). Public health implications of standardized 25-hydroxyvitamin D levels: A decrease in the prevalence of vitamin D deficiency among older women in Germany. Prev. Med..

[65] Bischoff-Ferrari H.A., Giovannucci E., Willett W.C., Dietrich T., Dawson-Hughes B. (2006). Estimation of optimal serum concentrations of 25-hydroxyvitamin D for multiple health outcomes. Am. J. Clin. Nutr..

[66] Kayaniyil S., Vieth R., Harris S.B., Retnakaran R., Knight J.A., Gerstein H.C., Perkins B.A., Zinman B., Hanley A.J. (2011). Association of 25(OH)D and PTH with metabolic syndrome and its traditional and nontraditional components. J. Clin. Endocrinol. Metab..

[67] Teixeira J.S., Bull Ferreira Campos A., Cordeiro A., Pereira S.E., Saboya C.J., Ramalho A. (2018). Vitamin D nutritional status and its relationship with metabolic changes in adolescents and adults with severe obesity. Nutr. Hosp..

